# Efficacy of Dapoxetine in the Treatment of Patients With Lifelong Premature Ejaculation as an Alternative to Sertraline Therapy

**DOI:** 10.1016/j.esxm.2021.100473

**Published:** 2021-12-27

**Authors:** Guoxiong Liu, Yinghao Yin, Lei Zhang, Dalin He, Lin Yang

**Affiliations:** 1 Department of Urology, the First Affiliated Hospital of Xian Jiaotong University, Xian, Shaanxi, P.R. China; 2 Department of Urology, the Third Xiangya Hospital of Central South University, Changsha, Hunan, P.R. China; 3 Department of Urology, Xijing Hospital of Air force Military Medical University, Xian, Shaanxi, P.R. China

**Keywords:** Dapoxetine, Sertraline, Premature ejaculation, Selective serotonin re-uptake inhibitors

## Abstract

**Introduction:**

Before dapoxetine was approved for the treatment of lifelong premature ejaculation (LPE) in China, daily dosing with off-label sertraline was common.

**Aim:**

To investigate the efficacy of dapoxetine in the treatment of patients with LPE as an alternative to sertraline therapy.

**Methods:**

This prospective study included LPE patients who previously attempted treatment with sertraline and who agree to receive dapoxetine therapy in our hospital from January 2020 to March 2021. Patients who received any PE therapy in the two months prior to the dapoxetine therapy were excluded. All patients received dapoxetine 30 mg (taken 1–3 hours before sexual intercourse) for 12 weeks, and they were not taking sertraline during the trial.

**Main Outcome Measure:**

Data on their intravaginal ejaculatory latency time and premature ejaculation profile were recorded before and after the dapoxetine treatment. Clinical Global Impression of Change scores and data on Treatment-Emergent adverse events were collected after treatment.

**Results:**

A total of 144 patients with LPE completed this study; including 64 patients who reported that previous sertraline treatment was satisfactory (group A) and 80 patients for whom previous sertraline therapy was unsatisfactory in treating PE (group B). Both groups experienced significantly increased intravaginal ejaculatory latency time. Dapoxetine therapy was reported satisfactory by 67.5% of patients with LPE in whom sertraline therapy unsatisfactory according to their Clinical Global Impression of Change score, which was not different from those who reported this result in group A (62.5%). Similar outcomes were also reported for premature ejaculation profile and treatment-emergent adverse events.

**Conclusion:**

: Although both dapoxetine and sertraline are selective serotonin re-uptake inhibitors, dapoxetine therapy is satisfactory in 67.5% of patients with LPE in whom sertraline treatment unsatisfactory, and the effect of dapoxetine was independent of the effect of sertraline.

**Liu G, Yin Y, Zhang L. et al., Efficacy of Dapoxetine in the Treatment of Patients With Lifelong Premature Ejaculation as an Alternative to Sertraline Therapy. Sex Med 2021;10:100473.**

## INTRODUCTION

Premature ejaculation (PE) is likely the most common male sexual dysfunction, affecting 21–39% of adult men worldwide and triggering sexual dissatisfaction, frustration, and distress among both them and their partners.^1^, ^2^, ^3^, ^4^ There are two primary types of PE: lifelong PE (LPE) and acquired PE (APE). LPE may be caused by specific genetic factors, while APE is often associated with endocrine, urological, and neurological factors or psychological issues.^5^^,^^6^ However, in either case, the etiology of PE is not definitively clear.

Treating PE with generic selective serotonin re-uptake inhibitors (SSRIs) has been confirmed to be safe and effective. Conventionally, these medications include paroxetine, fluvoxamine, fluoxetine, citalopram, and sertraline.^7^ Chronic daily treatment with SSRIs has been evaluated in numerous clinical trials for a variety of outcomes, especially intravaginal ejaculatory latency time (IELT) and adverse effects (AEs).^8^, ^9^, ^10^

Dapoxetine is a short-acting SSRI developed specifically for the treatment of PE.^11^^,^^12^ Unlike other SSRIs that require one week or longer achieving a steady-state concentration within the body, dapoxetine has a unique pharmacokinetic profile. After oral administration, it is rapidly absorbed (T_max_: 1.4–2.0 hours) and rapidly achieves peak plasma concentration (C_max_: 1–3 hours).^12^, ^13^, ^14^ The mean half-life of dapoxetine after a single dose was reportedly 1.3–1.5 hours, while the terminal half-life of dapoxetine was 15–19 h after a single dose.^12^^,^^13^ Moreover, dapoxetine has a dose-proportional pharmacokinetic profile, and its efficacy is not affected by food^14^. These characteristics and the drug's mild AEs suggest that dapoxetine is a good candidate for on-demand treatment of PE.

Before dapoxetine was approved by the Chinese Food and Drug Administration for treatment of PE in China, several SSRIs have been administered off-label in the treatment of PE, with sertraline being one of the most commonly prescribed drugs.^13^ If sertraline was unsatisfactory, another SSRI would instead be prescribed to patients with PE. However, both dapoxetine and sertraline are SSRIs, whether dapoxetine could be used in treating patients with LPE as an alternative to sertraline therapy?

## MATERIALS AND METHODS

### Patient Enrollment

This study was approved by the ethics committee of our hospital and registered online before patients’ enrollment. All subjects provided informed written consent prior to their inclusion in the study.

Eligible patients were enrolled from January 2020 to March 2021. All subjects were required to meet the following criteria: (1) men aged 20–64 years; (2) in a stable and heterosexual relationship for at least one year; (3) having regular sexual relations with a female partner during the study period; (4) experiencing LPE for at least one year; and (5) LPE diagnosed according to the definition of the International Society of Sexual Medicine's “Guidelines for the Diagnosis and Treatment of Premature Ejaculation,” in which LPE was defined as ejaculation always or nearly always occurring within approximately 1 minute of vaginal penetration ever since the first sexual experience.^14^ All eligible patients had to have previously received sertraline therapy for PE. All the patients should take at least 1 month of sertraline, and at least 4–6 times of intercourse per month, all the patients were asked to report their sertraline dose they had used for PE treatment.

Men who were considered to have APE, variable PE, or subjective PE were excluded. Additional exclusion criteria were: (1) erectile dysfunction or other forms of sexual dysfunction, penile diseases such as hypospadias and penile curvature deformity which may affect the sexual function; (2) a history of thyroid dysfunction, neurological disease, psychiatric disorder, or mental disorder; and (3) use of other forms of therapy for PE (pharmacological, behavioral or retardant condoms) in the two months prior to the study; (4) the effect of the sertraline were difficult to evaluated.

### Treatment and Assessment

The patients were evaluated for the effects of sertraline or dapoxetine prescribed for PE based upon patient satisfaction; in this study we used the Clinical Global Impression of Change (CGIC) assessment. During the CGIC assessment, after medication, the patient compared his PE condition to his performance before the medication, classifying the difference according to the following scoring system: three points, much better; two points, better; one point, slightly better; zero points, no change; −1 point, slightly worse; −2 points, worse; or −3 points, much worse^15^. Those with one, two, and three point(s) on the CGIC scoring system were recorded as having received satisfactory treatment (group A), while those with −3, −2, −1, or 0 point(s) on the CGIC scoring system were recorded as having received unsatisfactory treatment (group B).

To examine the efficacy and safety of dapoxetine in LPE patients as an alternative to sertraline therapy, all subjects received 30 mg dapoxetine 1–3 hours before planned intercourse and were directed to drink more than 250 mL of water. All patients were required to attempt sexual intercourse at least 4–6 times per month, and they were not taking sertraline during the trial.

Each patient reported their IELT and PEP at weeks 0, 4, and 12 and completed the CGIC at week 12 during the visit. AEs were reported spontaneously by patients when they occurred and were actively solicited by the study investigator. IELT was measured by a partner-held stopwatch and reported, while the CGIC and AEs were self-assessed by the patient. The CGIC was set as the primary outcome, IELT, PEP and AEs were secondary outcomes. All the questionnaires and scores used were validated in the Chinese.

### Statistical Analysis

Continuous variables were reported as mean ± standard deviation values, and the Kolmogorov–Smirnov test was used to test the normality of each variable. The IELT was presented in the form of geometric mean ± standard deviation values. Paired group comparisons of the baseline characteristics, between before and after the treatment were performed with a paired t-test. Categorical variables were expressed as frequencies (proportions) and compared using the chi-squared test. All analyses were performed using the Statistical Package for the Social Sciences version 13 software (IBM Corporation, Armonk, NY, USA). A two-sided value of *P* < .05 was considered statistically significant.

## RESULTS

A total of 171 patients with LPE who had previously received sertraline therapy were eventually enrolled in this study. Of these 171 patients, 83.1% of patients in group A (64/77) and 85.1% of patients in group B (80/94) completed the treatment. About 13 patients in group A and 14 patients in group B were lost during the follow-up, we had tried to contact these patients, most of them didn't answer the phone or they said they had difficult coming back and complete the questionnaire on time. The two groups were similar in terms of baseline demographics and clinical characteristics, including age, body mass index, IELT, and duration of PE; the one exception to this stratification was the dose of sertraline (Table 1), which was higher in group B.

**Table 1:** Baseline demographics and clinical characteristics (untreated)

	Group A	Group B	*P*-value
Total subjects, n	64	80	
Age, y	31.72 ± 6.38	32.24 ± 7.42	.36
BMI, kg/m^2^	22.32 ± 4.93	21.87 ± 5.38	.28
Duration of PE, y	4.16 ± 4.42	5.01 ± 4.66	.18
Sertraline dose			
50 mg	45 (70.3%)	19 (23.8%)	.01
100 mg	15 (23.4%)	33 (41.2%)	.01
>100 mg	4 (6.3%)	28 (35%)	.02
IELT, min	0.68 ± 0.31	0.64 ± 0.28	.78

BMI = body mass index; PE = premature ejaculation; IELT = intravaginal ejaculatory latency time.

In our hospital, all the PE patients treated with sertraline were initiated with dose 50 mg, if it was satisfactory, we will record it as sertraline satisfactory at dose 50 mg, if the patients reported dose 50 mg was not satisfied the dose will increased step by step until he was satisfied. Therefore, in group A, 70.3% (45/74) of PE patients with sertraline treatment stopped at dose 50 mg, they were satisfied with dose 50 mg; 23.4% (15/64) of PE patients stopped at dose 100 mg, and 6.4% (4/64) of PE patients stopped at dose 150 or 200 mg. And if the patients reported dose 50 mg was unsatisfied, we will record it as sertraline unsatisfactory at dose 50 mg, or if the patients still want to go ahead, we will increase the dose step by step. Therefore, 23.8% (19/84) of PE patients reported sertraline dose 50 mg were unsatisfied and not willing to go ahead, and these patients were recorded as sertraline unsatisfactory at dose 50 mg; 41.2% (33/84) of PE patients stopped at dose 100 mg and they didn't want to go ahead. About 35% (28/84) PE patients stopped at dose 150 and 200 mg, and they were recorded sertraline unsatisfactory at dose >100 mg. We found if sertraline 50 mg was unsatisfied, in most of PE patients they were sertraline unsatisfied even the dose increased. In few patients they were unsatisfied at low dose but satisfied at higher dose, these patients were not included in this study because they were difficult to evaluate.

Medication in both groups was associated with a significantly longer average IELT compared with to the baseline (Group A, increase from 0.68 to 2.84 minute, *P* = .02; Group B, increase from 0.64 to 2.71 minute; *P* = .01), the IELT at all follow-ups did not differ significantly between the two groups. (Table 2). While 40 (62.5%) patients in group A and 54 (67.5%) in group B reported CGIC scores of one, two, or three point(s) (slightly better, better, or much better) after therapy (*P* = .35).

**Table 2:** Intravaginal ejaculatory latency time (IELT) before and after treatment with dapoxetine in patients with lifelong premature ejaculation who previously attempted treatment with sertraline

	Week 0	Week 4	Week 12	*P*-value
Group A	0.68 ± 0.31	2.68 ± 0.82	2.84 ± 0.86	.02
Group B	0.64 ± 0.28	2.59 ± 0.78	2.71 ± 0.92	.01
*P*-value	.78	.82	.75	

In both groups, the PEP improved significantly. Significant improvements in perceived control (Group A: *P* = .02; Group B: *P* = .03) and satisfaction with sexual intercourse (Group A: *P* = .02; Group B: *P* = .02) were apparent beginning at week 4, and these improvements were maintained through week 12. After dapoxetine treatment, both groups showed significant improvements in ejaculation-related personal distress (Group A: *P* = .04; Group B: *P* = .03) and interpersonal difficulty (Group A: *P* = .04; Group B: *P* = .04) beginning at week 4 and continuing through the study endpoint, with no differences between the two groups (Table 3). AEs of dapoxetine in both groups were compared, including dizziness, nausea, headache, diarrhea, and fatigue (Table 4). There was no difference between the two groups.

**Table 3:** Premature ejaculation profile (PEP) before and after treatment with dapoxetine in patients with lifelong premature ejaculation who previously attempted treatment with sertraline

		Week 0	Week 4	Week 12	*P*-value
Perceived control	Group A	0.67 ± 0.21	1.71± 0.42	1.84 ± 0.36	.02
	Group B	0.64 ± 0.28	1.59 ± 0.48	1.71 ± 0.32	.03
	*P*-value	.78	.82	.72	
Satisfaction with sexual intercourse	Group A	0.45 ± 0.21	1.58 ± 0.38	1.72 ± 0.26	.02
	Group B	0.41 ± 0.19	1.69 ± 0.32	1.81 ± 0.29	.02
	*P*-value	.71	.42	.61	
Ejaculation-related personal distress	Group A	3.41 ± 0.61	2.54 ± 0.52	2.34 ± 0.56	.04
	Group B	3.49 ± 0.68	2.49 ± 0.58	2.21 ± 0.51	.03
	*P*-value	.77	.53	.58	
Interpersonal difficulty	Group A	2.83 ± 0.51	2.23 ± 0.39	2.18 ± 0.31	.04
	Group B	2.84 ± 0.48	2.29 ± 0.38	2.11 ± 0.37	.04
	*P*-value	.82	.80	.71

**Table 4:** Incidence of adverse effects after dapoxetine treatment in patients with lifelong premature ejaculation

	Group A	Group B	*P-*value
Total subjects, n	64	80	
Dizziness, n (%)	6 (9.5%)	8 (10%)	.22
Nausea, n (%)	4 (6.3%)	6 (7.7%)	.18
Headache, n (%)	2 (3.2%)	4 (5.0%)	.14
Diarrhea, n (%)	2 (3.2%)	3 (3.8%)	.24
Fatigue, n (%)	2 (3.2%)	3 (3.8%)	.24

## DISCUSSION

PE is thought to be the most common male sexual dysfunction. Due to the intimate nature of the problem, most patients hesitate to seek treatment.^16^^,^^17^ PE largely affects the patient's sexual confidence and their intimate relationships, as well as the sexual satisfaction of both them and their partner.^18^^,^^19^

In the absence of an approved treatment for PE, conventional SSRIs are widely used. Daily treatment of 50–200 mg of sertraline is effective for PE. Ejaculation delay usually occurs within 5–10 days of starting the treatment, but it may take two weeks for the full therapeutic effect to manifest; ejaculation delay is usually maintained with long-term use. However, daily use of sertraline may cause unwanted and prolonged side effects,^8^, ^9^, ^10^ such as dizziness, nausea, headache, diarrhea, and fatigue, and, given its off-label nature, the use of sertraline for the treatment of PE is considered risky in China.

Dapoxetine is a potent SSRI with rapid absorption and elimination. Dapoxetine was found to increase the IELT by 2.5–3 minute, with limited and tolerable side effects. It has been approved as an on-demand treatment option for PE in many countries, including China.^11^^,^^20^ Dapoxetine has been evaluated to date in several large, randomized, double-blind, placebo-controlled phase 3 trials that have included more than 6,000 men from more than 25 countries. In these trials, a significant increase in IELT was found to be associated with dapoxetine treatment, beginning with the first dose and maintained at all subsequent time points.^14^^,^^20^, ^21^, ^22^, ^23^ Among patients receiving 30 mg of dapoxetine, 30.7% reported longer IELT and CGIC scores that were better or much better, as compared with 13.9% of placebo-treated patients.^22^ Our results are consistent with previous observations in other countries.^21^, ^22^, ^23^ These data indicate that dapoxetine is effective for PE treatment in Chinese patients.

In the process of treating PE, there is no single SSRI that is effective for all patients with PE; so, if one SSRI fails, another SSRI is trialed. However, was the second SSRI to be effective or not, or what percentage of the second SSRI would be effective have not been widely considered.

In this study, we proved that, among patients with LPE in whom sertraline treatment unsatisfactory, dapoxetine treatment was satisfactory for 67.5% of the patients, which indicated that, despite unsatisfactory of sertraline treatment, dapoxetine could still be prescribed for the treatment of these patients with PE. When taken after full elution, 62.5% of patients with LPE, in whom sertraline is satisfactory, reported dapoxetine to be satisfactory, and there were no significant differences between the two groups. This means that the effect of dapoxetine to treat LPE is independent of the satisfaction of sertraline.

It is well known that SSRIs can block axonal re-uptake of serotonin [5-hydroxytryptamine (5-HT)] from the synapse through 5-HT transporters, enhance 5-HT neurotransmission, stimulate postsynaptic membrane 5-HT receptors, and eventually result in ejaculatory delay.^7^^,^^8^ Both dapoxetine and sertraline are SSRIs, and their mechanism of action is almost the same; however, the structure varies between the two drugs; the pharmacokinetics was different, and we presumed receptor selective and bioavailability may explain these clinical differences in satisfactory. Further research is needed on how this structural dissimilarity causes the difference in their effects on PE.

In this study, we used CGIC to evaluate the satisfaction of dapoxetine and sertraline for the treatment of LPE. CGIC is a self-evaluation assay based on patients’ subjective feelings; so, it is not as objective a measure as IELT and may be influenced by physical or psychological factors. However, somehow, PE itself is a kind of subjective concept based on the PE diagnostic criteria^14^, and we should understand that patients’ satisfaction is our primary treatment target, especially among patients with PE. Therefore, we used the CGIC as the standard to evaluate the PE therapeutic effect.

It was not surprising that the TEAEs were similar between the two groups since patients in both groups were taking the same medicine at the same dose. We proved that the TEAEs of dapoxetine were independent of sertraline's effect. However, most of our documented AEs, such as nausea, diarrhea, headache, dizziness, insomnia, and somnolence, were transient, mild, and easy to tolerate; thus, dapoxetine is a safe medicine for Chinese patients with LPE.

Our study is limited by its small patient number, short follow-up time, and the use of a single dose of dapoxetine. The absence of a placebo group are other relevant limitations, however, for a study Initiated by researchers, we could not get a standard placebo without the supported by the company. Although we proved that dapoxetine could be used for patients with LPE in whom sertraline treatment unsatisfactory, we could not explain the mechanism behind this effect.

## CONCLUSIONS

Although both dapoxetine and sertraline are SSRIs, dapoxetine therapy was satisfactory for 67.5% of patients with LPE in whom sertraline treatment previously unsatisfactory. The effect of dapoxetine was independent of the effect of sertraline.

## ACKNOWLEDGMENT

We thank Accdon (www.accdon.com) for its linguistic assistance and scientific consultation during the preparation of this manuscript.

## STATEMENT OF AUTHORSHIP

Lin Yang and Dalin He designed and performed the experiments, analyzed the data, and drafted the manuscript. Yinghao Yin designed the study and prepared the manuscript. Guoxiong Liu and Lei Zhang performed the study. All authors approved the final version of the manuscript.
